# Uptake of Gold Nanoparticles in Several Rat Organs after Intraperitoneal Administration *In Vivo*: A Fluorescence Study

**DOI:** 10.1155/2013/353695

**Published:** 2013-07-17

**Authors:** Mohamed Anwar K. Abdelhalim

**Affiliations:** Department of Physics and Astronomy, College of Science, King Saud University, P.O. Box 2455, Riyadh 11451, Saudi Arabia

## Abstract

*Background*. The gold nanoparticles (GNPs) have potential applications in cancer diagnosis and therapy. In an attempt to characterise the potential toxicity or hazards of GNPs as a therapeutic or diagnostic tool, the fluorescence spectra in several rat organs *in vivo* were measured after intraperitoneal administration of GNPs. *Methods*. The experimental rats were divided into control and six groups (G1A, G1B, G2A, G2B, G3A, and G3B; G1: 20 nm; G2: 10 nm; G3: 50 nm; A: infusion of GNPs for 3 days; B: infusion of GNPs for 7 days). The fluorescence measurements were investigated in the liver, kidney, heart, and lung organs of rats after intraperitoneal administration of GNPs for periods of 3 and 7 days *in vivo*. *Results*. The 10 and 20 nm GNPs exhibited spherical morphology shape, while the 50 nm GNPs exhibited hexagonal shape. A sharp decrease in the fluorescence intensity induced with the larger 50 nm GNPs in the liver, kidney, heart, and lung organs of rats at the exposure duration of 3 and 7 days *in vivo* compared with the smaller 10 and 20 nm GNPs was observed. *Conclusions*. The decrease in fluorescence intensity may be attributed to occurrence of strong quenching, decrease in number and surface area of GNPs, and high clearance of GNPs via urine and bile. Moreover, decreasing size may lead to an exponential increase in surface area relative to volume, thus making GNPs surface more reactive on aggregation and to its surrounding biological components. The size, shape, surface area, number, and clearance of GNPs play a key role in toxicity and accumulation in the different rat organs. This study demonstrates that fluorescence peak intensity is particle size and exposure duration dependent. This study suggests that fluorescence intensity can be used as a useful tool for pointing to bioaccumulation and toxicity induced by GNPs in the different rat organs.

## 1. Introduction

Fluorescence is the emission of light by a substance that has absorbed light or other electromagnetic radiation of a different wavelength. In most cases, the emitted light has a longer wavelength, and therefore lower energy, than the absorbed radiation. However, when the absorbed electromagnetic radiation is intense, it is possible for one electron to absorb two photons; this two-photon absorption can lead to emission of radiation having a shorter wavelength than the absorbed radiation. Fluorescence has many practical applications, including mineralogy, chemical sensors, dyes, and biological detectors [1].

Nanotechnology has recently emerged as a promising field for the treatment and diagnosis of a variety of diseases [2]. GNPs are particularly promising because of their ease of synthesis in various shapes and the potential for conjugation with peptides and proteins, which can target the GNPs to specific interaction partners [3].

The origin of the unique optical properties of GNPs is a phenomenon known as surface plasmon resonance (SPR). When an electromagnetic radiation, of a wavelength much smaller than the diameter of the GNPs, hits the particles, it induces coherent, resonant oscillations of the metal electrons across the NPs. These oscillations are known as the SPR, which lie within visible frequencies and result in strong optical absorbance and scattering properties of the GNPs [4, 5]. This property allows the use of GNPs for many applications, as Raman sensors [6], photocatalysts [7], and photoelectrochemical materials [8, 9], while in the bioscience and medical fields, GNPs can be used as immunostaining marker for electron microscopy and as chromophores for immunoreactions and nucleic acid hybridization [10, 11]. 

Numerous studies have shown that exposure to smaller sized particles can produce inflammatory and cytotoxic effects when compared to exposure to larger sized particles at equivalent mass concentration [22]. It is considered that smaller NPs can be more reactive with biological components and have adverse effects due to large surface area and much particle number [23]. 

Toxicity has been thought to originate from nanomaterial size and surface area, composition, and shape. The nanoparticle size plays a role in how the body responds to, distributes, and eliminates materials [7, 11]. The particle size can also affect the mode of endocytosis, cellular uptake, and the efficiency of particle processing in the endocytic pathway [24, 25]. 

The GNPs show several features that make them well suited for biomedical applications, including straightforward synthesis, stability, and the potential for surface modification with active biological molecules such as peptides or proteins [26]. Semmler-Behnke et al. [27] observed that a considerable percentage of 18 nm GNPs is removed from the blood and trapped predominantly in the liver and spleen. 

The GNPs can be used in various biomedical applications; however, very little is known about their particle size and exposure duration dependence *in vivo*. The GNPs can offer great promise for biomedical applications, particularly in novel diagnostic and therapeutic methods [2]. Here, we focus our attention on aspects related to fluorescence spectroscopy for different GNP sizes dissolved in aqueous solution and at different exposure duration periods in several rat organs *in vivo*. Moreover, the GNPs size and shape are monitored by the transmission electron microscopy (TEM). 

## 2. Materials and Methods

### 2.1. Gold Nanoparticles (GNPs)

The 10, 20, and 50 nm GNPs (products MKN-Au-010, MKN-Au-020, and MKN-Au-050, Canada, resp.) were purchased. All GNPs used in this study were in aqueous solution at a concentration of 0.01%. The mean size and morphology of these GNPs were evaluated using transmission electron microscope (TEM) images. Moreover, the homogeneity of GNPs in terms of shape and size was evaluated. 

### 2.2. Animals

Healthy male Wistar-Kyoto rats were obtained from the Laboratory Animal Centre (College of Pharmacy, King Saud University). Rats of age 8–12 weeks (approximately 250 g body weight) were housed in pairs in humidity- and temperature-controlled ventilated cages on a 12 h day/night cycle. A conventional rodent diet and water were provided. Forty rats were individually caged and divided into a control group (NG: *n* = 10), group 1 (A: infusion of 20 nm GNPs for 3 days; *n* = 5; B: infusion of 20 nm GNPs for 7 days; *n* = 5), group 2 (A: infusion of 10 nm GNPs for 3 days; *n* = 5; B: infusion of 10 nm GNPs for 7 days; *n* = 5), and group 3 (A: infusion of 50 nm GNPs for 3 days; *n* = 5; B: infusion of 50 nm GNPs for 7 days; *n* = 5). Doses (50 *μ*L) of 10, 20, or 50 nm GNPs in aqueous solution were administered to the animals via intraperitoneal injection every day for 3 or 7 days. The rats were anesthetised by inhalation of 5% isoflurane until muscular tonus relaxed. Liver, kidney, heart, and lung organs were dissected and collected from each rat. In order to assess rat organ uptake, as much blood as possible was collected from the rats to maximise residual blood drainage from the organs. All experiments were conducted in accordance with guidelines approved by the King Saud University Local Animal Care and Use Committee.

### 2.3. Digestion of Rat Organ Samples

Liver, kidney, heart, and lung samples were dissected from each rat, wet-digested with nitric acid, and stored as acidic digest solutions for analysis by fluorescence spectroscopy. The rat organs were first freeze-dried to minimize and to facilitate subsequent sample preparation steps. Each sample organ was then homogenised to a fine powder by ball milling in plastic containers. Approximately 0.20–0.25 g of powdered sample organ was weighed into a Teflon reaction vessel and 3 mL of HNO_3_ was added. The closed reaction vessel was heated in a 130°C oven until digestion was completed. The rat organ samples were then diluted to a final volume of 20 mL with quartz distilled water and stored in 1 oz. polyethylene bottles for subsequent fluorescence spectroscopy analysis.

### 2.4. Fluorescence Spectroscopy

Fluorescence spectra for several rat organs after intraperitoneal administration of 10, 20, or 50 nm GNPs and for periods of 3 and 7 days were obtained using a FluoroMax-2 spectrofluorometer (JOBIAN YVON-SPEX, Instruments S. A., Inc., France). Fluorescence measurements were made over the wavelength range 250–700 nm using 1 cm path length quartz cuvettes, which were cleaned before each use by sonicating for 5 min in deionised water and then rinsing with deionised water.

### 2.5. Histological Investigation

Fresh portions of liver, heart, kidney, and lung from each rat were cut rapidly, fixed in neutral buffered formalin (10%), and then dehydrated, with grades of ethanol (70, 80, 90, 95, and 100%). Dehydration was then followed by clearing the samples in 2 changes of xylene. Samples were then impregnated with 2 changes of molten paraffin wax, and then embedded and blocked out. Paraffin sections (4-5 *μ*m) were stained with hematoxylin and eosin (the conventional histological and stain) according to [15–17]. Stained sections of control and treated rats were examined for alterations in the hepatocytes for the presence of inflammatory, fatty change and Kupffer cells hyperplasia and necrosis [13–15]. 

## 3. Results and discussion

### 3.1. Size and Morphology of Different GNPs

**Figure 1:**
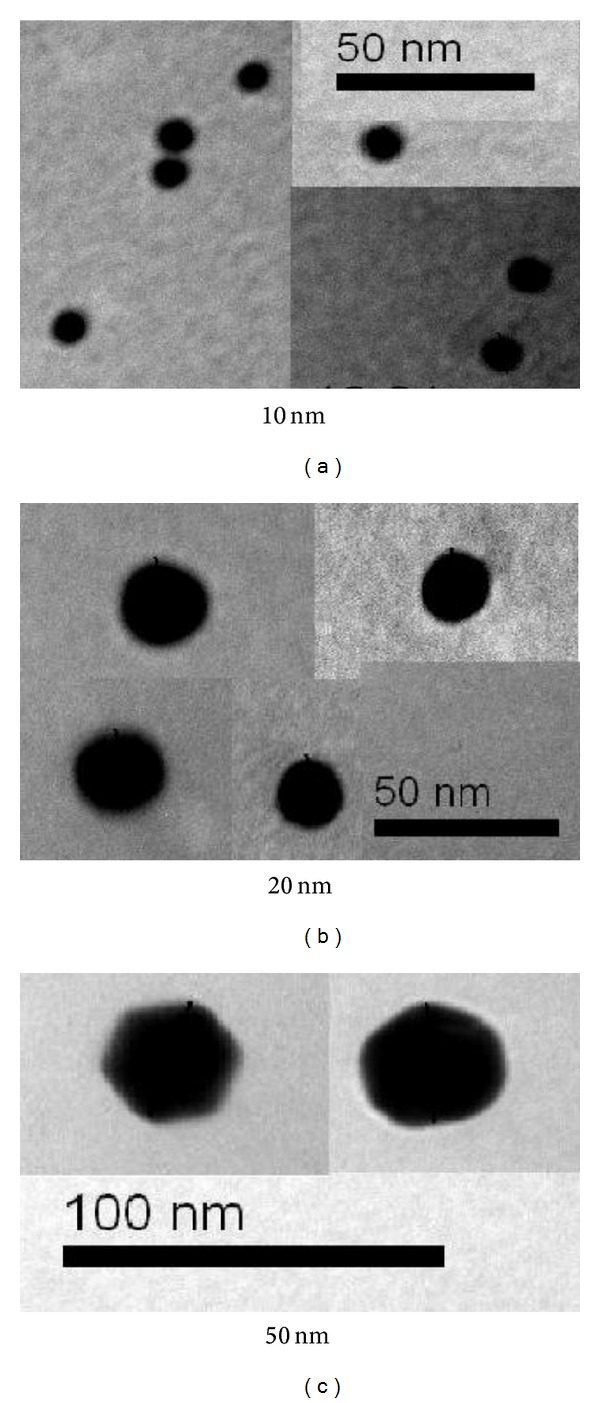
TEM images for different GNP samples [12].

**Figure 2:**
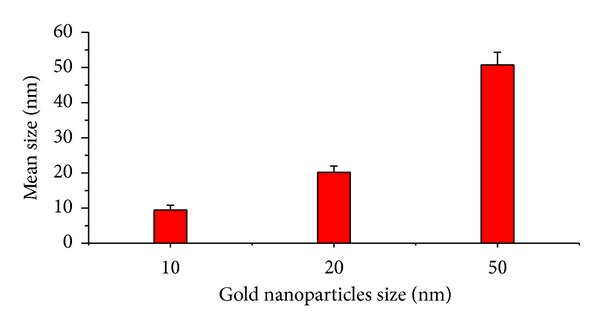
The size of 10, 20, and 50 nm gold nanoparticles measured using TEM images [12].

The 10 and 20 nm GNPs showed spherical morphology with a narrow particle size distribution when dispersed in solution. The mean size for these GNPs was calculated from the TEM images. The mean measured size was 9.45 ± 1.33 nm for 10 nm GNPs and 20.18 ± 1.80 nm for 20 nm GNPs. GNPs of 50 nm diameters, in contrast, were not spherical but hexagonal in TEM images, as shown in Figures 1 and 2. The high electron density and homogeneous shape and size of the GNPs make them highly conspicuous under the TEM [12].

**Figure 3:**
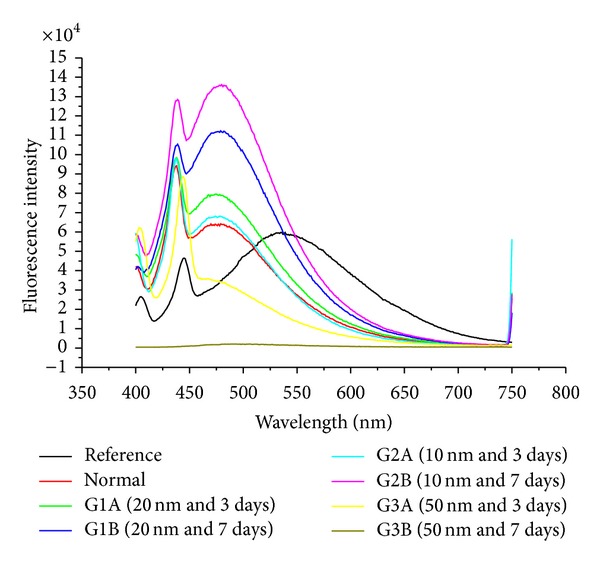
The liver fluorescence emission peak intensities after intraperitoneal administration of 10, 20, and 50 nm GNPs for periods of 3 and 7 days.

The size effect of GNPs at the infusion periods of 3 days (G1A, G2A, and G3A) and 7 days (G1B, G2B, and G3B) on the fluorescence intensity of the liver organ is shown in Figure 3. The fluorescence peak intensity increased for G1A, G2A, G1B, and G2B and decreased for G3A and G3B compared with the control. The fluorescence peak intensity for G1B was higher than G1A and for G2B than G2A [12, 28–30]. This result demonstrates that the fluorescence intensity is GNPs size and exposure duration dependent.

**Figure 4:**
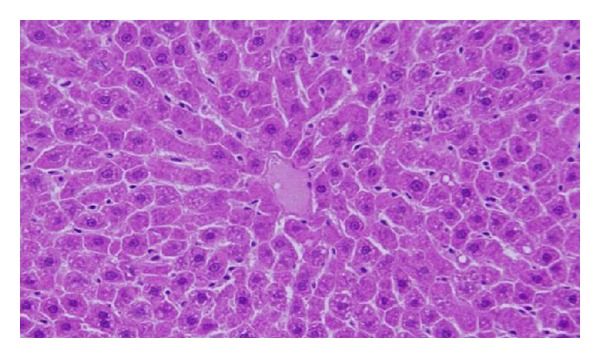
GNPs-normal rat demonstrated normal hepatocyte.

**Figure 5:**
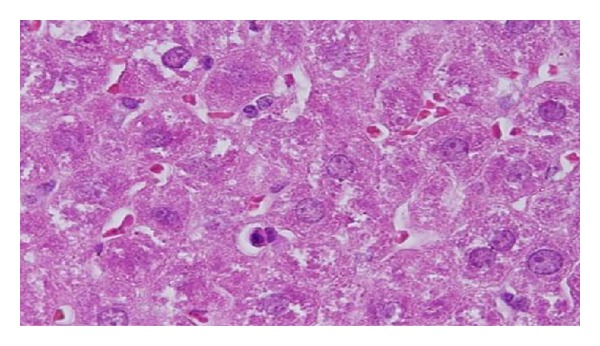
GNPs-treated rat received 50 *μ*L of 10 nm GNPs for 3 days [13–15].

GNPs-normal rat demonstrated that normal hepatocyte is shown in Figure 4. Cloudy swelling with pale cytoplasm and poorly delineated and displaced nuclei in all GNPs-treated rats observed (Figure 5). The ballooning degeneration was more prominent with 10 nm size particles than the larger ones. This swelling might be exhibited as a result of disturbances of membranes function leads to massive influx of water and Na^+^ due to GNPS effects. Cellular swelling might be accompanied by leakage of lysosomal hydrolytic enzymes that lead to cytoplasmic degeneration and macromolecular crowding [13–15]. The vacuolated swelling of the cytoplasm of the hepatocytes of the GNPs-treated rats might indicate acute and subacute liver injury induced by these NPs. Variable nuclei sizes were observed in some hepatocytes. 

The GNPs-normal rat demonstrated normal hepatocyte (Figure 4). The GNPs-treated rat received 50 *μ*L of 10 nm GNPs for 3 days demonstrating hepatocytes cloudy swelling [13–15] (Figure 5).

**Figure 6:**
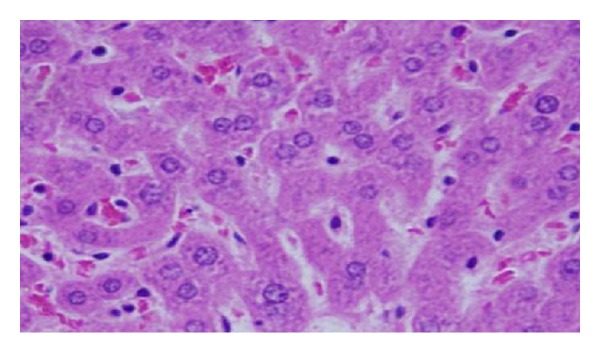
GNPs-treated rat received 50 *μ*L of 10 nm particles for 7 days [13–15].

The sinusoidal Kupffer cells became prominent and increased in number due to GNPs exposure. This change was more prominent with 10 nm GNPs and dose of 100 *μ*L than 50 nm GNPS and more after 7 days of administration than rats exposed to GNPs for 3 days as shown in Figure 6. Kupffer cells activation might indicate that GNPs activate the phagocytic activity of the sinusoidal cells by increasing the number of kupffer cells to help in removing the accumulated GNPs where lysosomes are involved in the intracellular breakdown into small metabolic products. The produced Kupffer cells hyperplasia might be correlated with the amount of injurious to the hepatic tissue induced by GNPs intoxication and represent a defense mechanism of detoxification. Kupffer cell hyperplasia contributes to hepatic oxidative stress [13, 14].

**Figure 7:**
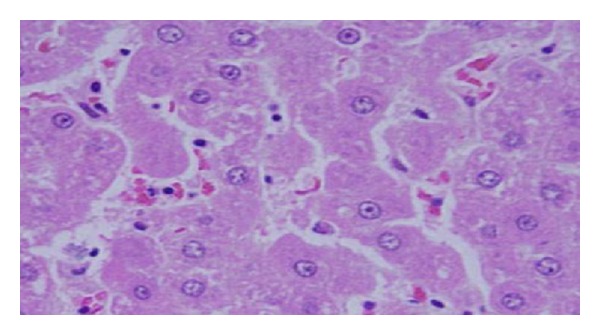
GNPs-treated rat received 50 *μ*L of 10 nm particles for 3 days [13–15].

Sporadic spotty well-defined necrosis was noticed in some hepatocytes of GNPs-treated rats. The insulted cells exhibited highly eosinophilic amorphous cytoplasm with occasional apoptotic characterization (Figure 7). This alteration was detected in the liver of rats exposed to 10 nm size GNPs but was not seen with those exposed to 50 nm size GNPs. Apoptotic alteration might be followed by organelles swelling, specially mitochondria, endoplasmic reticulum, and rupture of lysosomes which might lead to amorphous eosinophilic cytoplasm as an initial sign in the sequence of hepatocytes necrosis before shrinking and dissolution of nuclei [13, 14]. The seen hepatocytes necrosis due to GNPs exposure might indicate oxidative stress on these cells by glutathione depletion [16, 17].

**Figure 8:**
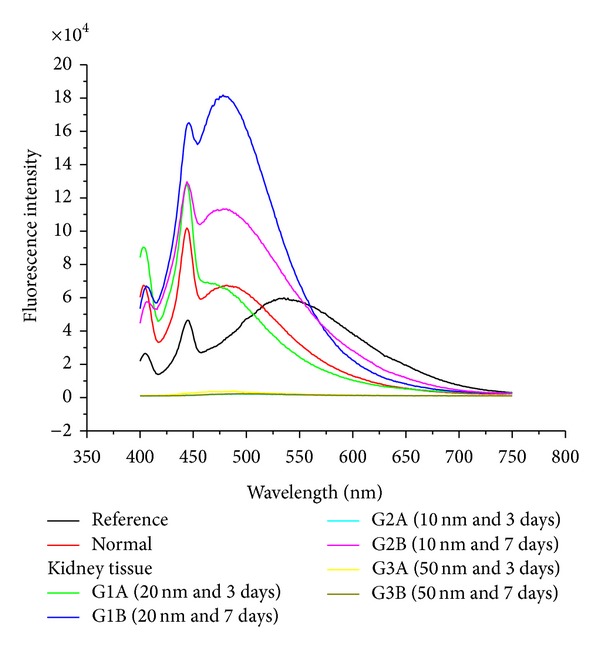
The kidney fluorescence emission peak intensities after intraperitoneal administration of 10, 20, and 50 nm GNPs for periods of 3 and 7 days.

The size effect of GNPs at the infusion periods of 3 days (G1A, G2A, and G3A) and 7 days (G1B, G2B, and G3B) on the fluorescence intensity of the kidney organ is shown in Figure 8. The fluorescence peak intensity increased for G1A, G2A, G1B, and G2B and decreased for G3A and G3B compared with the control. The fluorescence peak intensity for G1B was higher than G1A and for G2B than G2A [12, 28–30]). This result demonstrates that the fluorescence intensity is GNPs size and exposure duration dependent. 

**Figure 9:**
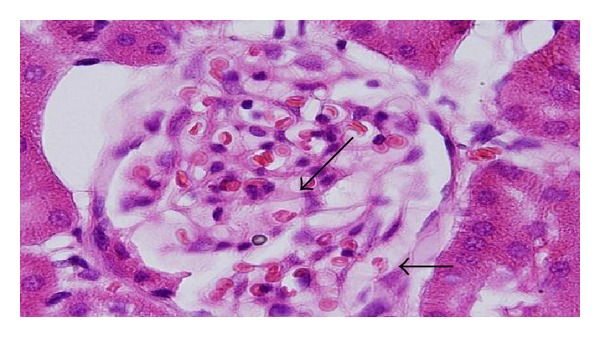
GNPs-treated rat received 50 *μ*L of 10 nm particles for 3 days [16, 17].

**Figure 10:**
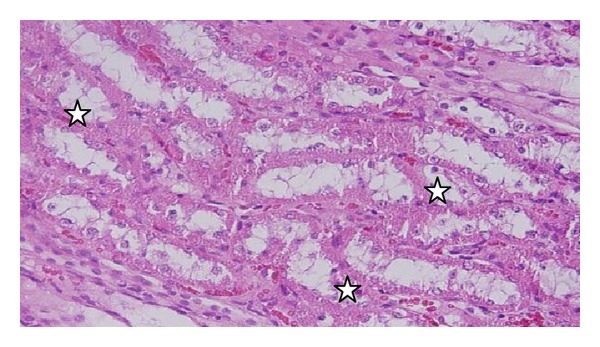
GNPs-treated rat received 50 *μ*L of 10 nm particles for 3 days [16, 17].

**Figure 11:**
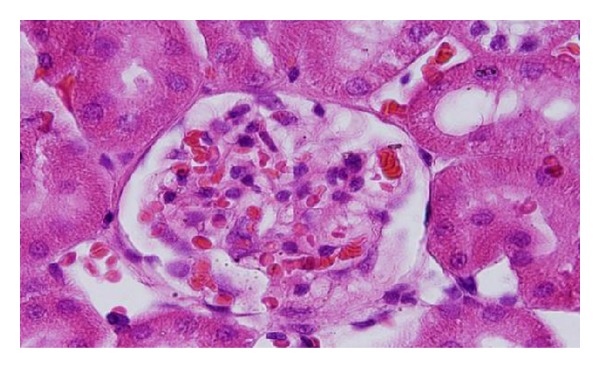
GNPs-treated rat received 50 *μ*L of 10 nm particles for 3 days [16, 17].

The GNPs-treated rat which received 50 *μ*L of 10 nm particles for 3 days demonstrated glomerular congestion (arrow) [18, 19] (Figure 9). The GNPs-treated rat which received 50 *μ*L of 10 nm particles for 3 days demonstrated vacuolar degeneration stars): vacuolization of the renal cells was seen and increased in severity in the renal tubules of rats which received 100 *μ*L of 10 or 20 nm GNPs with less or no vacuolar degeneration with 50 nm particles. More vacuolar degeneration was observed in the renal cells of rats exposed to 7 days than ones exposed to 3 days [18, 19] (Figure 10). The GNPs-treated rat which received 50 *μ*L of 10 nm particles for 3 days demonstrated hyaline droplets in the cytoplasm of the renal cells (arrows). Hyaline droplets were detected in the renal epithelium of rats which received 100 *μ*L of 10 or 20 nm GNPs. Droplets appearance is associated with protein metabolism disturbances. This alteration was not seen in the renal tissue of rats exposed to 50 nm particles [18, 19] (Figure 11).

**Figure 12:**
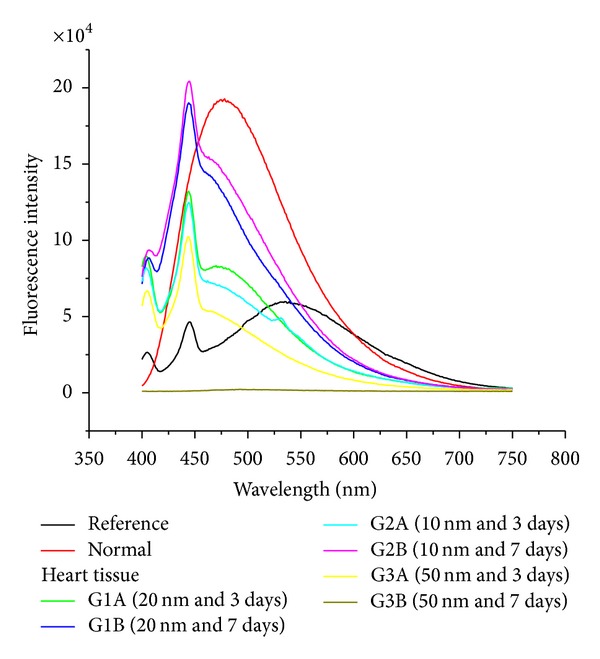
The heart fluorescence emission peak intensities after intraperitoneal administration of 10, 20, and 50 nm GNPs for periods of 3 and 7 days.

The size effect of GNPs at the infusion periods of 3 days (G1A, G2A, and G3A) and 7 days (G1B, G2B, and G3B) on the fluorescence intensity of the heart organ is shown in Figure 12. The fluorescence peak intensity increased for G1A, G2A, G1B, and G2B and decreased for G3A and G3B compared with the control. The fluorescence peak intensity for G1B was higher than G1A and for G2B than G2A [12, 28–30]. This result demonstrates that the fluorescence intensity is GNPs size and exposure duration dependent. 

**Figure 13:**
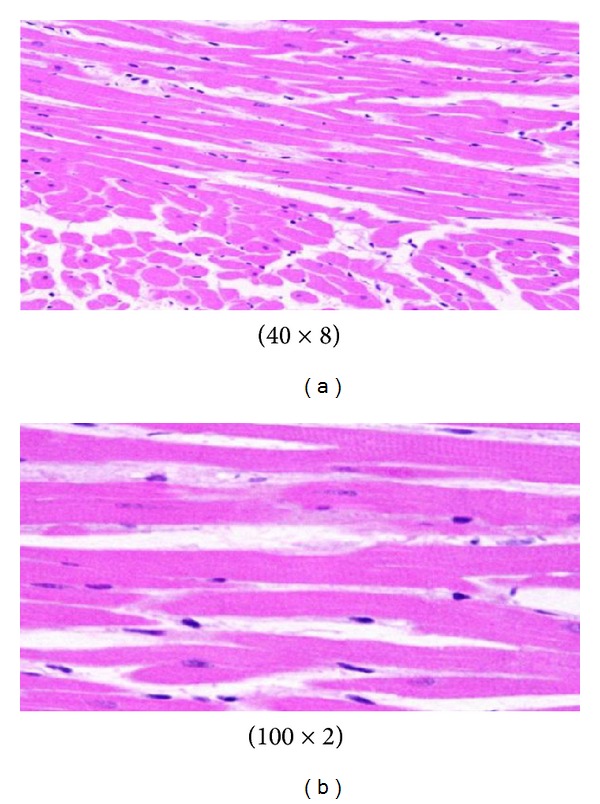
Microscopic pictures show GNPs-normal rats [18, 19].

**Figure 14:**
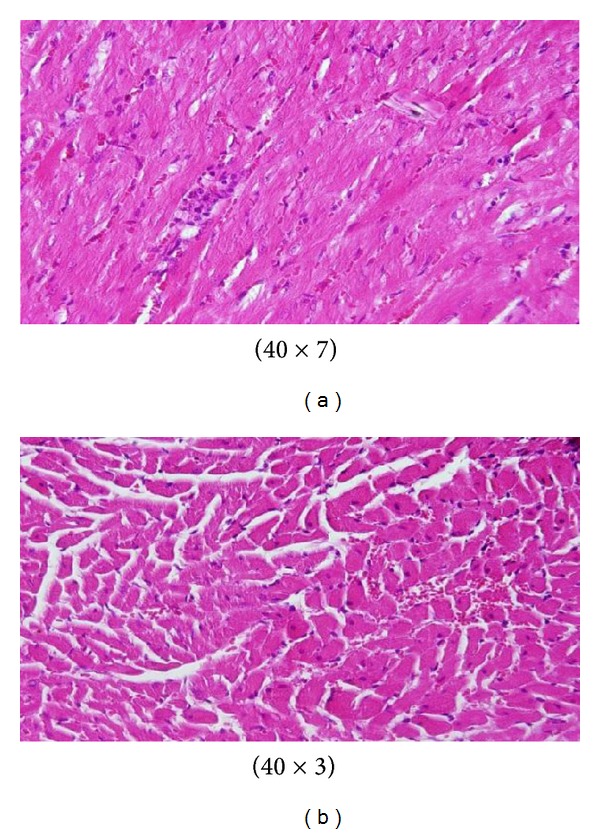
GNPs-treated rat received 50 *μ*L of 10 nm particles for 3 days [18, 19].

**Figure 15:**
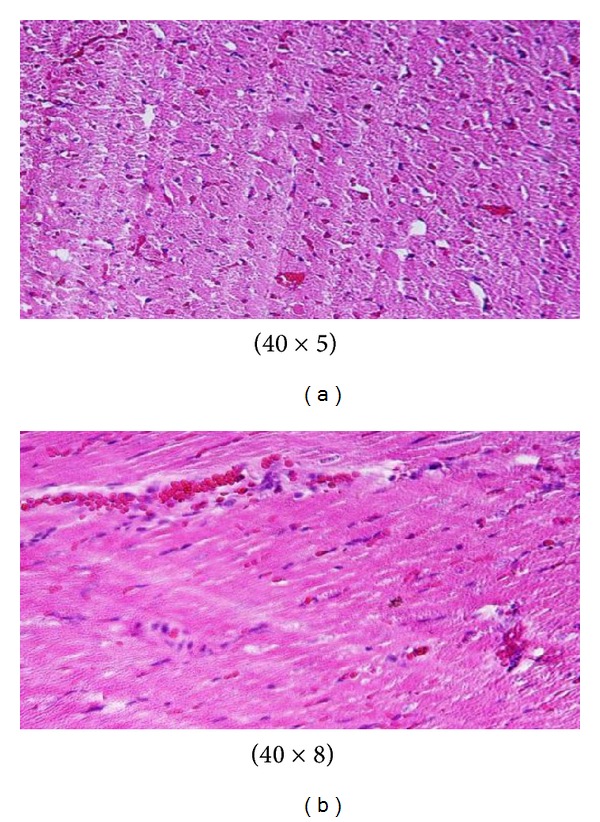
GNPs-treated rat received 50 *μ*L of 10 nm particles for 7 days [18, 19].

Microscopic pictures show that GNPs-normal rat demonstrated benign, blunt-looking heart muscle with various heart muscle orientations and with no pathological findings [18, 19] (Figure 13). The GNPs-treated rat which received 50 *μ*L of 10 nm particles for 3 days demonstrated extravasation of red blood cells with few scattered lymphocytic infiltrate [18, 19] (Figure 14). The GNPs-treated rat which received 50 *μ*L of 10 nm particles for 7 days demonstrated scattered foci of hemorrhage with extravasation of red blood cells, a few scattered cytoplasmic vacuolization [18, 19] (Figure 15).

**Figure 16:**
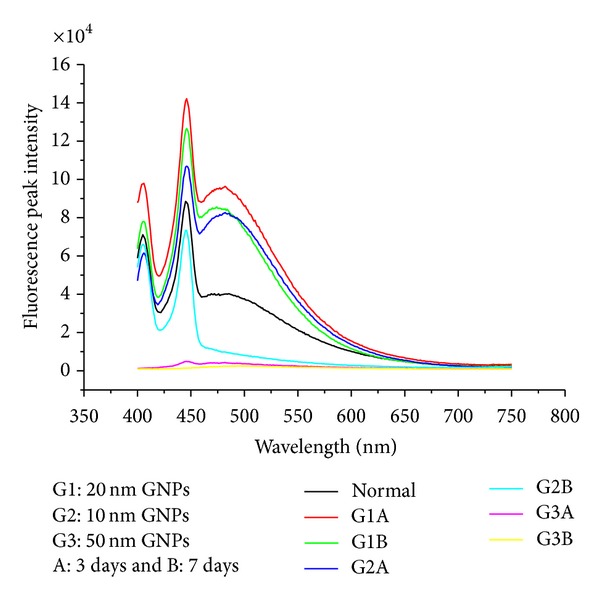
The lung fluorescence emission peak intensities after intraperitoneal administration of 10, 20 and 50 nm GNPs for periods of 3 and 7 days.

The size effect of GNPs at the infusion periods of 3 days (G1A, G2A, and G3A) and 7 days (G1B, G2B, and G3B) on the fluorescence intensity of the lung organ is shown in Figure 16. The fluorescence peak intensity increased for G1A, G2A, and G1B and decreased for G2B, G3A, and G3B compared with the control. The fluorescence peak intensity for G1A was higher than G1B, and for G2A than G2B [12, 28–30]. This result demonstrates that the fluorescence intensity is GNPs size and exposure duration dependent. 

**Figure 17:**
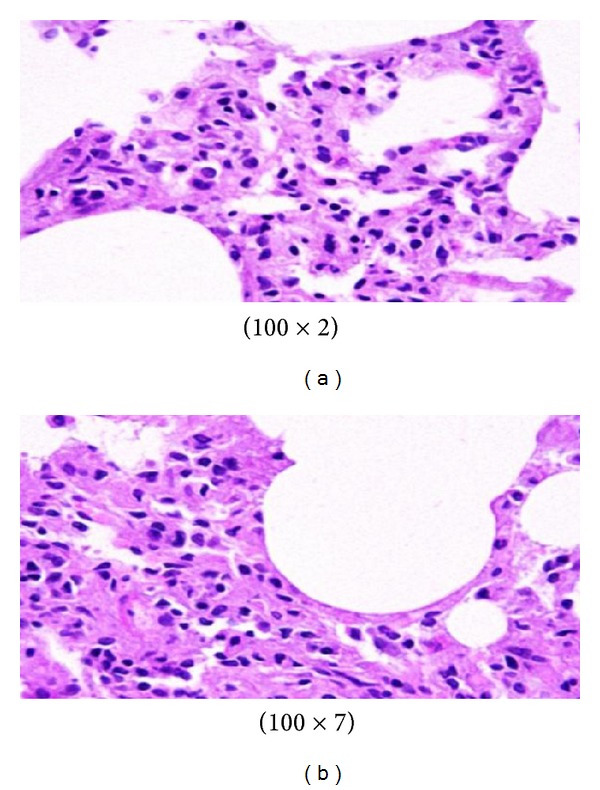
Control group: microscopic pictures show GNP-normal rats [20, 21].

**Figure 18:**
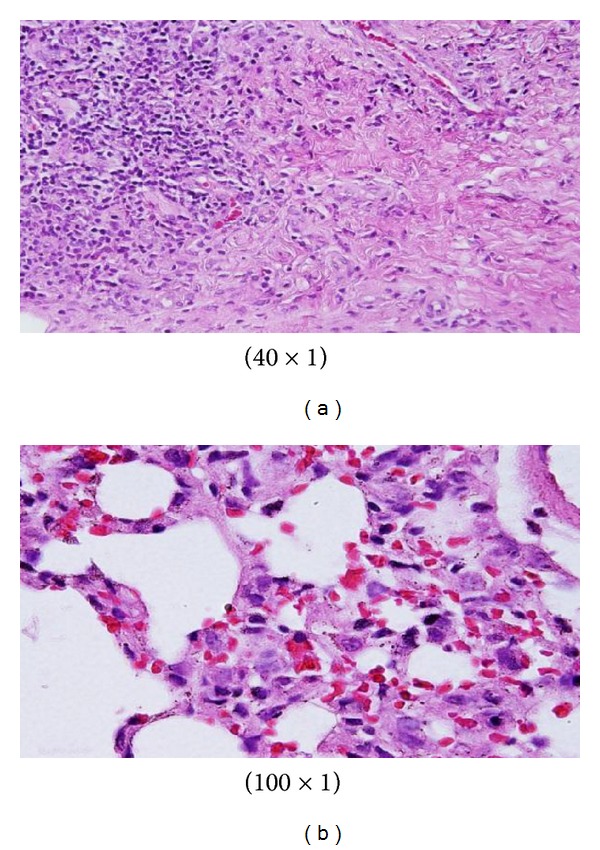
GNPs-treated rats received 50 *μ*L of 10 nm GNPs for 3 days [20, 21].

**Figure 19:**
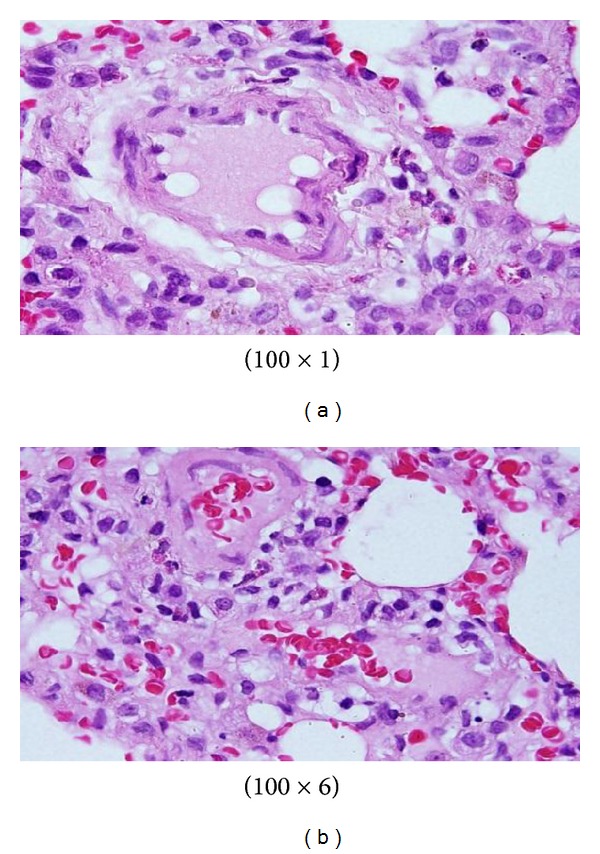
GNP-treated rats received 50 *μ*L of 10 nm particles for 7 days [20, 21].

Control group: microscopic pictures show that GNP-normal rats demonstrated well-formed and open alveoli with normal spate, few scattered small lymphocytes, and minimal eosinophils [20, 21] (Figure 17). The GNPs-treated rats which received 50 *μ*L of 10 nm GNPs for 3 days demonstrated more diffuse interstitial pneumonia, dense inflammatory cell infiltrates of small lymphocytes, fibrosis, and more prominent extravasation of red blood cells [20, 21] (Figure 18). The GNP-treated rats which received 50 *μ*L of 10 nm particles for 7 days demonstrated prominent chronic inflammatory cell infiltrates surrounded by dilated and congested blood vessels, scattered dense extravasation of red blood cells, and foci of hemosiderin granules [20, 21] (Figure 19).

**Figure 20:**
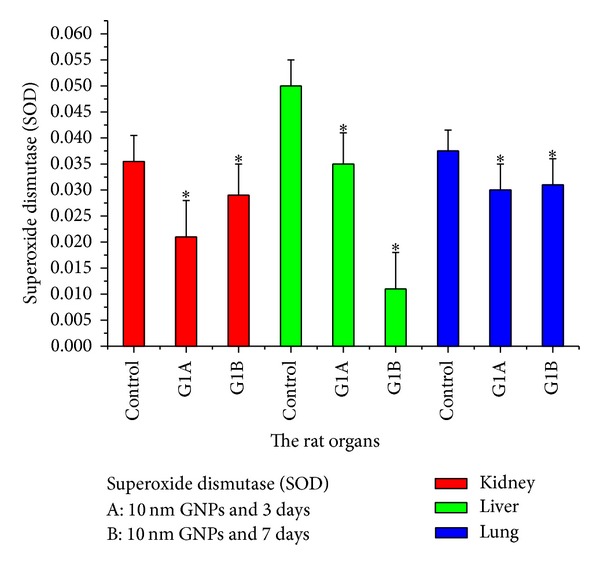
The superoxide dismutase (SOD) levels after intraperitoneal administration of 10 nm GNPs for exposure duration of 3 (G1A) and 7 (G1B) days in the rat kidney, liver, and lung organs.

The level of superoxide dismutase (SOD) significantly decreased in rat kidney liver and lung organs after intraperitoneal administration of 10 nm GNPs for exposure duration of 3 and 7 days compared with the control (Figure 20). The SOD which catalyzes the dismutation of the superoxide anion (O_2_^∙−^) into hydrogen peroxide and molecular oxygen is one of the most important antioxidative enzymes. 

**Figure 21:**
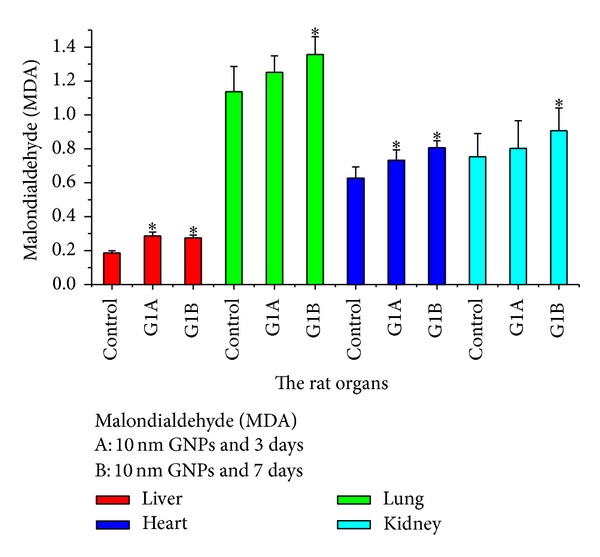
The malondialdehyde (MDA) levels after intraperitoneal administration of 10 nm GNPs for exposure duration of 3 (G1A) and 7 (G1B) days in the rat liver, lung, heart, and kidney organs.

The malondialdehyde (MDA: lipid peroxidation) values significantly increased in rat liver, lung, heart, and kidney organs after intraperitoneal administration of 10 nm GNPs for exposure duration of 3 and 7 days compared with the control (Figure 21). This indicates the increased production of free radicals or ROS in these organs as a result of intraperitoneal administration of GNPs into rats, concomitant with the increased production of MDA. Haseeb Khan et al. [31] found the same result for MDA in rat liver organ. The smaller particles tend to be more toxic than the larger ones. The exposure of GNPs (average diameter 5.3 ± 1 nm) produced oxidative stress within 24 h in mytilusedulis [32, 33]. 

The organ distributions of GNPs are size dependent, while small GNPs of 5–15 nm have wider organ distribution than that of large GNPs of 50–100 nm [27, 34, 35]. It has been found that GNPs with a long blood circulation time can accumulate in the liver and spleen and significantly affect the gene expression [36]. Thus, the hepatotoxicity of GNPs may be attributed to accumulation of NPs in liver.

The liver and spleen are considered two dominant organs for biodistribution and metabolism of GNPs [27, 34–36]. If GNPs are larger than renal filtration cutoff, they are not excreted in urine; instead they are eliminated from the blood by the reticuloendothelial system and thus tend to accumulate in the spleen and liver [34, 37]. 

The decrease in fluorescence intensity observed with the larger 50 nm GNPs compared with the smaller 10 and 20 nm GNPs may be attributed to the following factors: (1) formation of a strong ground state complex between serum albumins and GNPs (static quenching); (2) differences in the physical and chemical properties of nanoparticles of different sizes and shapes (the 50 nm GNPs were hexagonal, and nanoparticle properties are highly size and shape dependent); (3) faster uptake of and clearance by liver macrophages of the 50 nm GNPs compared to the other nanoparticles.

The fluorescence peak intensity in the liver, kidney, and heart organs of rats for G1B was higher than G1A and for G2B than G2A. This result demonstrates that the fluorescence intensity is GNPs size and exposure duration dependent, while the fluorescence peak intensity in the lung organ of rats for G1A was higher than G1B and for G2A than G2B. This result demonstrates that the fluorescence intensity is GNPs size and exposure duration dependent. 

The results of this study indicate that decrease in GNPs size produces an exponential increase in surface area relative to volume, which may make the GNPs more self-reactive (i.e., may promote aggregation) and more prone to interactions with surrounding molecules (biological components). Moreover, increased uptake of NPs may lead to accumulation in certain tissues, where the particles may interfere with critical biological functions [9, 24]. 

The smaller nanoparticles size imparts physical and chemical properties that are very different from those of the same material in bulk form. They have a larger surface area to volume ratio compared to bulk materials; they may thus exhibit an enhanced or hindered tendency to aggregate (depending on the surface chemistry), enhanced photoemission, high electrical or heat conductivity, or improved surface catalytic activity [22, 24]. 

The nanoparticle surfaces can interact with biological components, and nanoparticles may be more reactive than larger particles toward biomolecules. It has been shown, for example, that the severity and the likelihood of inflammatory response transiently increased, within 12 h, following injection of 200 or 100 nm GNPs into experimental animals. GNPs were ultimately trapped by macrophages in the spleen and liver and remained in these tissues until 4 weeks after the single injection [10].

The present results suggest that the larger 50 nm GNPs may be highly cleared via urine and bile. Nanoparticles for therapeutic use need to have a long retention time in order to encounter and interact with the desired target. However, a long retention time can result in toxic effects *in vivo*. Thus, route and rate of nanomaterial clearance is an important issue [38]. The absorbed nanoparticles in the systemic circulation can be excreted through various routes, such as the kidneys or bile. Renal clearance of solid nanosized materials is known to be influenced by particle size and surface charge [38, 39].

The smaller 10 and 20 nm GNPs have shown a propensity to accumulate in the several rat organs following injection. The rat organs distribution of GNPs was size and exposure duration dependent; the smaller GNPs showed the most widespread organ accumulation and distribution. 

## 4. Conclusions

The aim of the present study was to evaluate the fluorescence spectra of several rat organs after intraperitoneal administration of 10, 20, and 50 nm GNPs at exposure duration of 3 and 7 days *in vivo* in an attempt to characterise the potential toxicity or hazards of GNPs as a therapeutic tool.

High electron density and homogeneous shape and size make GNPs highly conspicuous in TEM images. The 10 and 20 nm GNP exhibited spherical morphology shape, while the 50 nm GNPs exhibited hexagonal shape. 

A sharp decrease in fluorescence intensity induced with the larger 50 nm GNPs in the liver, kidney, heart, and lung organs of rat *in vivo* was observed. This decrease may be attributed to occurrence of strong quenching, and decrease in number and surface area of GNPs in addition to high clearance of GNPs via urine and bile. Moreover, the decrease in size may lead to an exponential increase in surface area relative to volume, thus making GNPs surface more reactive on itself (aggregation) and to its surrounding environment (biological components). 

This study demonstrates that fluorescence peak intensity is particle size and exposure duration dependent. The size, shape, surface area, number, and clearance of GNPs play a key role in toxicity, and the distribution and accumulation of GNPs in the different rat organs may be mediated by dynamic protein binding and exchange. This study suggests that fluorescence intensity may be used as a useful and important diagnostic tool for the distribution and bioaccumulation induced by the administration of GNPs to rats.
